# Supplementary material for: Analysis of PPARγ Signaling Activity in Psoriasis
Source: Int J Mol Sci. 2021 Aug 10;22(16):8603. doi: 10.3390/ijms22168603 (PMC8395241; doi:10.3390/ijms22168603)
Supplement: Supplementary file 1 [file ijms-22-08603-s001.zip › Supplemental materials_Analysis of PPARg signaling activity in psoriasis/Pathway models/Models images and html files/Model of PPARG signaling in psoriasis/37594.html]

NAD(P)H oxidase


# Functional Class NAD(P)H oxidase

|  |  |
| --- | --- |
| URN | urn:agi-aopfc:0000479 |
| Total Entities | 3 |
| Connectivity | 5577 |
| Name | NAD(P)H oxidase |
| Description | NADPH oxidase |

---

|  |  |
| --- | --- |
| ChildConcepts | cytochrome b558 |
|  | TC 5.B.1.2 |
|  | NOX4 |

---

|  |  |
| --- | --- |
| Pathway | mTOR Signaling Activation by Fatty Acids and Glucose |
|  | Hypoxia Inhibits mTOR Signaling |
|  | Protein Oxidation and Aggregation in Aging |
|  | AMPK Related Catabolism Deceleration in Glucose Insufficiency |
|  | Lipoxin A4/FPR2-Related Neutrophil Depression |
|  | Neutrophil Activation via LTB4/Polyisoprenyl Phosphate Signaling |
|  | Eosinophil Activation |
|  | Macrophage M1 Lineage |
|  | CD16/CD14 Proinflammatory Monocyte Activation |
|  | Vascular Endothelial Cell Activation by NO |
|  | GFs/TNF -> Ion Channels |
|  | Nociception Expression Targets Signaling |
|  | EDNRA/B -> Vascular Motility |
|  | ADRA1A -> IL6 Production |
|  | ADRA1 -> Prostaglandin Generation |
|  | BDKRB1/2 -> Prostaglandin Generation |
|  | HTR1 -> IL6 Production |
|  | HTR5 -> TNF Production |
|  | HTR1 -> Membrane Transport |
|  | Scavenger Receptor OLR1 in Inflammation Related Endothelial Dysfunction |
|  | Dilated Cardiomyopathy |
|  | Neurons Necrosis Caused by Energy Deficiency |
|  | Apoptotic Keratinocytes Clearance Recession in Systemic Lupus Erythematosus |
|  | Oxidative Stress in Vitiligo |
|  | Vitiligo |
|  | Toll-like Receptors in beta-Cell |
|  | beta-Cell Death in Diabetes Mellitus Type 2 |
|  | beta-Cell Recovery in Diabetes Mellitus |
|  | Hyperglycemia and Hyperlipidemia Trigger beta-Cell Apoptosis |
|  | Fatty Acids in Hypertension and Atherosclerosis Development |
|  | Eosinophil Activation and Degranulation in Asthma |
|  | CFTR Facilitates Oxidative Stress in Airway Epithelium in CF |
|  | Airway Epithelial Cell Dysfunction |
|  | Ca2+ Overload in Muscular Dystrophies |
|  | Muscular Dystrophies |
|  | Dystrophin Glycoprotein Complex Signaling in Muscular Dystrophies |
|  | Neurotoxin Effects in Parkinson's Disease |
|  | Brain Cell Necrosis in Vascular Dementia |
|  | Oxidative Stress, All-Trans-Retinal and Lipofuscin Toxicity in AMD |
|  | Alveolar Macrophages Dysfunction in COPD |
|  | Neutrophil Associated Lung Damage in Chronic Obstructive Pulmonary Disease |
|  | Penile Detumescense Augmentation Mechanisms |
|  | Advanced Glycation End Products in Diabetes or Age Related Erectile Dysfunction |
|  | Proteins with Altered Expression in Erectile Dysfunction |
|  | Proteins Involved in Erectile Dysfunction |
|  | Doxorubicin Induced Cardiotoxicity |
|  | Cyclosporine Induced Nephrotoxicity |
|  | ROS Metabolism |
|  | EGFR Transactivation in Cancer and Non-Cancer Cells |
|  | Asbestosis- and Silicosis-Associated Inflammation Provokes Cancer Development |
|  | Oxidized LDL/OLR1 Activates Cell Proliferation in Cancer |
|  | Respiratory Chain Impairment in Cancer |
|  | AGT -> TP53 Expression Targets |
|  | AGT -> CREB Expression Targets |
|  | AGT -> ELK/SRF Expression Targets |
|  | TNF-alpha/TNFRSF1A Signaling |
|  | Ras Signaling |
|  | HIF1 Signaling |
|  | PI3K/RAC1 Signaling |
|  | ROS in Neutrophil Mediated Cell Damage |
|  | ROS in Triggering Vascular Inflammation |
|  | ROS and RNS in Vasoconstriction and Vasodilation Regulation |
|  | Ca2+ Cytosolic Overload |
|  | ROS in Angiotensin Mediated Cardiovascular Remodeling and Hypertrophy |
|  | prarg neg,ukn expres targets, ps-positive |
|  | Neighbors of NAD(P)H oxidase |
|  | comon ps\_pos, pprarg\_neg targets |
|  | Model of PPARG signaling in psoriasis |
|  | PPARG negative regulators and targets |
|  | 2\_Chondrocyte death in osteoarthritis |
|  | 2\_Podocyte dysfunction in glomerulonephritis |
|  | 2\_Oxidative stress, all-trans-retinal, and lipofuscin toxicity in agerelated macular degeneration |
|  | 1\_Low-density lipoprotein internalization and the accumulation of lipids in atherosclerosis |
|  | 1\_Endothelial cells stimulate vasoconstriction and thrombosis in pulmonary hypertension |
|  | 2\_Hyperglycemia and hyperlipidemia trigger beta-cell apoptosis |
|  | 3\_2\_Beta-cell proliferation and compensation: neogenesis/dedifferentiation |
|  | 3\_Pain perception in diabetic neuropathy |
|  | 2\_Neuron and Schwann cell death |

---

|  |  |
| --- | --- |
| MedScan ID | 12811045 |

---

|  |  |
| --- | --- |
| Alias | NAD(P)H oxidase complexes |
|  | NADP(H) oxidases |
|  | ThOXs |
|  | nicotinamide adenine dinucleotide phosphate oxidases |
|  | thyroid oxidase |
|  | ThOX |
|  | dual oxidases |
|  | NADPH oxidase complexes |
|  | thyroid NADPH oxidase |
|  | ThOX/DUOX |
|  | respiratory burst oxidase |
|  | superoxide-generating NADPH oxidases |
|  | Rbohs |
|  | superoxide-generating NADPH oxidase |
|  | DUOX |
|  | NADPH oxidase |
|  | neutrophil cytochrome B |
|  | NADH/NADPH oxidases |
|  | NAD(P)H:oxygen oxidoreductase |
|  | cytochrome b-245 |
|  | NADH/NADPH oxidase |
|  | NADPH:oxygen oxidoreductase |
|  | flavoprotein NAD(P)H oxidase |
|  | NAD(P)H oxidase |
|  | NADH/NAD(P)H oxidase |
|  | superoxide-generating NADPH oxidase activity |
|  | NADPH oxidases |
|  | flavoprotein NADPH oxidase |
|  | DUOXes |
|  | Rboh |
|  | superoxide-generating NADP(H) oxidase |
|  | NADP(H) oxidase |
|  | nicotinamide adenine dinucleotide phosphate oxidase |
|  | NAD(P)H oxidases |
|  | respiratory burst oxidase homolog |
|  | respiratory burst oxidase homologs |
|  | reduced nicotinamide adenine dinucleotide phosphate oxidases |
|  | respiratory burst oxidases |
|  | NADH/NAD(P)H oxidases |
|  | respiratory burst oxidase homologue |
|  | 1.6.3.1 |
|  | flavoprotein NAD(P)H oxidases |
|  | superoxide-generating NAD(P)H oxidase |
|  | thyroid oxidases |
|  | thyroid NAD(P)H oxidase |
|  | dual oxidase |
|  | thyroid NADPH oxidases |
|  | DUOXs |
|  | NAD(P)H oxidase complex |
|  | NADP(H) oxidase complex |
|  | NADPH oxidase complex |
|  | superoxide-generating NAD(P)H oxidases |
|  | NADPH:oxygen oxidoreductases |
|  | thyroid NADP(H) oxidases |
|  | superoxide-generating NAD(P)H oxidase activity |
|  | thyroid NADP(H) oxidase |
|  | thyroid NAD(P)H oxidases |
|  | superoxide-generating NADP(H) oxidase activity |
|  | superoxide-generating NADP(H) oxidases |
|  | flavoprotein NADP(H) oxidases |
|  | flavoprotein NADP(H) oxidase |
|  | flavoprotein NADPH oxidases |
|  | cytochrome b558 |
|  | flavocytochrome b558 |
|  | NADP(H):oxygen oxidoreductases |
|  | NADP(H):oxygen oxidoreductase |
|  | NAD(P)H:oxygen oxidoreductases |
|  | NADH/NADP(H) oxidase |
|  | NADH/NADP(H) oxidases |
|  | Neutrophil cytochrome B |

---

|  |  |
| --- | --- |
| EC Number | 1.6.3.1 |
|  | 1.6.9.91 |

---

|  |  |
| --- | --- |
| GO ID | 0016175 |

---

|  |  |
| --- | --- |
| Cell Localization | Cytoplasm |

---
